# Use of steroids to treat anti-tumor necrosis factor α induced tuberculosis-associated immune reconstitution inflammatory syndrome: Case report and literature review

**DOI:** 10.1097/MD.0000000000022076

**Published:** 2020-10-23

**Authors:** Daijiro Nabeya, Takeshi Kinjo, Kazutaka Yamaniha, Shoshin Yamazato, Reo Tome, Kazuya Miyagi, Hideta Nakamura, Tetsu Kinjo, Shusaku Haranaga, Futoshi Higa, Jiro Fujita

**Affiliations:** a Department of Infectious, Respiratory, and Digestive Medicine, Graduate School of Medicine, University of the Ryukyus; b Department of Respiratory Medicine, Okinawa Chubu Hospital; c Department of Respiratory Medicine, National Hospital Organization Okinawa National Hospital, Okinawa, Japan.

**Keywords:** corticosteroid, immune reconstitution inflammatory syndrome, infliximab, TNFα antagonist, tuberculosis

## Abstract

**Introduction:**

Individuals with tuberculosis (TB) who are being treated with anti-tumor necrosis factor α (anti-TNFα) for coexisting conditions may experience unexpected exacerbations of TB after the initiation of antituberculous therapy, so-called anti-TNFα-induced TB-immune reconstitution inflammatory syndrome (anti-TNFα-induced TB-IRIS). Anti-TNFα-induced TB-IRIS is often treated empirically with corticosteroids; however, the evidence of the effectiveness of corticosteroids is lacking and the management can be a challenge.

**Patient concerns:**

A 32-year-old man on long-term infliximab therapy for Crohn disease visited a clinic complaining of persistent fever and cough that had started 1 week previously. His most recent infliximab injection had been administered 14 days before the visit. A chest X-ray revealed a left pleural effusion, and he was admitted to a local hospital.

**Diagnosis:**

A chest computed tomography (CT) scan revealed miliary pulmonary nodules; acid-fast bacilli were found in a sputum smear and a urine sediment sample; and polymerase chain reaction confirmed the presence of *Mycobacterium tuberculosis* in both his sputum and the pleural effusion. He was diagnosed with miliary TB.

**Interventions:**

Antituberculous therapy was started and he was transferred to our hospital for further management. His symptoms initially improved after the initiation of antituberculous therapy, but 2 weeks later, his symptoms recurred and shadows on chest X-ray worsened. A repeat chest CT scan revealed enlarged miliary pulmonary nodules, extensive ground-glass opacities, and an increased volume of his pleural effusion. This paradoxical exacerbation was diagnosed as TB-IRIS associated with infliximab. A moderate-dose of systemic corticosteroid was initiated [prednisolone 25 mg/day (0.5 mg/kg/day)].

**Outcomes:**

After starting corticosteroid treatment, his radiological findings improved immediately, and his fever and cough disappeared within a few days. After discharge, prednisolone was tapered off over the course of 10 weeks, and he completed a 9-month course of antituberculous therapy uneventfully. He had not restarted infliximab at his most recent follow-up 14 months later.

**Conclusion:**

We successfully managed a patient with anti-TNFα-induced TB-IRIS using moderate-dose corticosteroids. Due to the limited evidence currently available, physicians should consider the necessity, dosage, and duration of corticosteroids for each case of anti-TNFα-induced TB-IRIS on an individual patient-by-patient basis.

## 1 Introduction

Anti-tumor necrosis factor α-induced tuberculosis immune reconstitution inflammatory syndrome (anti-TNFα-induced TB-IRIS) was first described in 2005.^[1]^ IRIS is a well-known phenomenon in the treatment of human immunodeficiency virus (HIV). HIV-associated IRIS is defined as the worsening of inflammatory symptoms during the immune reconstitution induced after starting antiretroviral therapy. Increased antigen levels, due to the death of *Mycobacterium tuberculosis* in response to antituberculous therapy, can trigger TB-IRIS in patients with HIV.^[2]^

Although the incidence of anti-TNFα-induced TB-IRIS (4/56, 7%)^[3]^ appears to be lower than that of HIV-associated TB-IRIS (median: 18%, range: 4–54%),^[4]^ anti-TNFα-induced TB-IRIS may be triggered by a mechanism similar to that of HIV-associated TB-IRIS. The pathogenic mechanism of anti-TNFα-induced TB-IRIS is unknown. However, it may occur as a result of reconstituted immune responses against *M. tuberculosis* causing an uncontrolled inflammatory reaction.^[5]^

Appropriate management of anti-TNFα-induced TB-IRIS is a challenge.^[6]^ Previous case reports have shown that anti-TNFα-induced TB-IRIS may respond to high-dose corticosteroids.^[1]^ However, there is currently a lack of consensus regarding the use and dosage of corticosteroids for the treatment of anti-TNFα-induced TB-IRIS. Here, we report a case of anti-TNFα-induced TB-IRIS that was controlled using a moderate dose of systemic corticosteroids. In addition, we review published case reports of anti-TNFα-induced TB-IRIS and discuss its treatment, focusing on the use of corticosteroids. This study was reviewed and approved by the Clinical Research Ethics Committee of University of the Ryukyus.

## 2 Case report

A 32-year-old man who had been diagnosed with Crohn disease 7 years previously was admitted to our hospital with TB. Since diagnosis, his Crohn disease had been managed with infliximab. *Before starting infliximab,* he had tested negative for *M. tuberculosis* using an interferon-γ release assay. He visited a clinic complaining of persistent fever and cough that had started one week previously. His most recent infliximab injection had been administered 14 days before the visit. A chest X-ray revealed a left pleural effusion, and he was admitted to a local hospital. A computed tomography (CT) scan of his chest revealed miliary pulmonary nodules; acid-fast bacilli were found in a sputum smear and a urine sediment sample; and polymerase chain reaction confirmed the presence of *M. tuberculosis* in both his sputum and the pleural effusion. He was diagnosed with miliary TB and was started on antituberculous therapy (isoniazid, rifampicin, pyrazinamide, and ethambutol). Six days after starting antituberculous therapy, he was transferred to the University of the Ryukyus Hospital in Okinawa, Japan, for further management.

**Figure 1:**
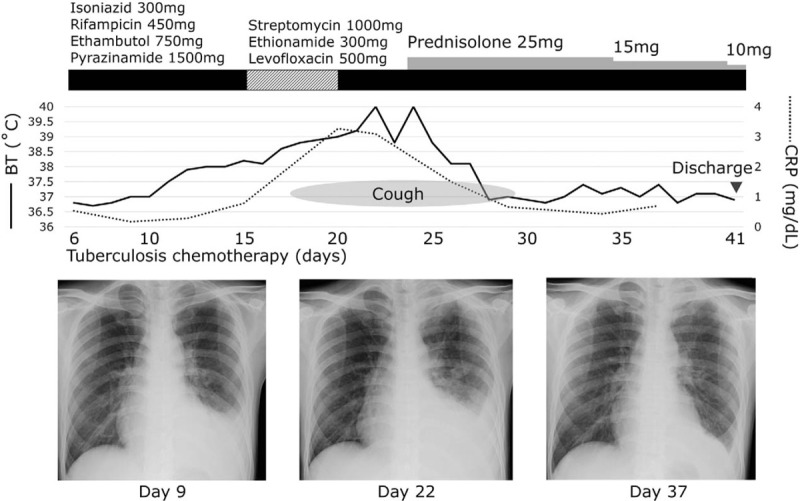
Clinical course and chest X-ray images of the patient. The patient experienced clinical deterioration about two weeks after starting antituberculous therapy. All clinical manifestations of TB-IRIS rapidly resolved after starting systemic corticosteroids.

**Figure 2:**
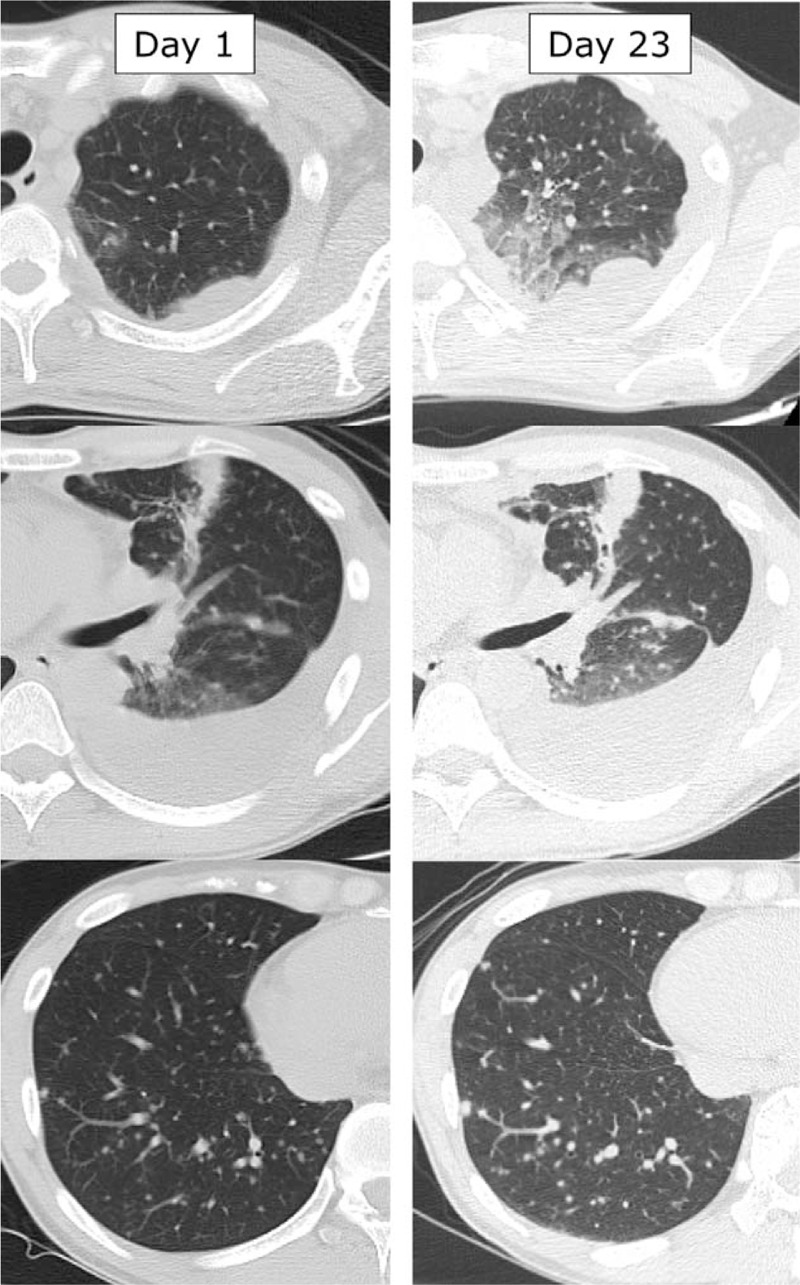
Computed tomography scans of the patient's chest. Day 1: CT scan on the day of starting antituberculous therapy. Day 23: CT scan 23 days after starting antituberculous therapy. The images show newly formed ground-glass opacities, increased pleural effusion on the left side, and enlarged miliary pulmonary nodules.

The patient's clinical course and chest X-ray findings are shown in Figure 1. His symptoms initially improved after starting antituberculous therapy, but his high fever recurred within 2 weeks of starting treatment, and his cough recurred within 3 weeks. Initially, his fever was thought to be a reaction to the antituberculous drugs, so his TB treatment regimen was changed to a combination of streptomycin, ethionamide, and levofloxacin on Day 15. However, his clinical condition continued to deteriorate. A repeat chest CT scan, done on Day 23, revealed enlarged miliary pulmonary nodules, extensive ground-glass opacities, and an increased volume of his pleural effusion (Fig. 2). This paradoxical exacerbation was diagnosed as anti-TNFα-induced TB-IRIS, associated with *i*nfliximab, and a systemic corticosteroid was initiated [prednisolone 25 mg/day (0.5 mg/kg/day)] on Day 24. After starting corticosteroid treatment, his radiological findings improved immediately, and his fever and cough disappeared within a few days. He was discharged on Day 41.

After discharge, the *M. tuberculosis* strain that had been cultured from a sputum sample was found to be fully susceptible to the antituberculous drugs that were being administered. Prednisolone was tapered off over the course of 10 weeks, and he completed a 9-month course of antituberculous therapy uneventfully. Infliximab was withdrawn at the initiation of antituberculous therapy and had not been restarted at the time of his most recent follow-up, 14 months after his discharge.

The patient provided written informed consent for his case history, and chest X-ray and CT scans to be published (University of the Ryukyus Hospitals consent form).

## 3 Literature review and discussion

We searched for previous case reports of anti-TNFα induced TB-IRIS using PubMed, and found reports on 21 additional cases. The details of all 22 cases, including the one presented here, are summarized in Table 1.^[1,7–22]^ In all cases, TNFα antagonists were initially withdrawn after anti-TNFα-induced TB-IRIS was diagnosed.

**Table 1:**
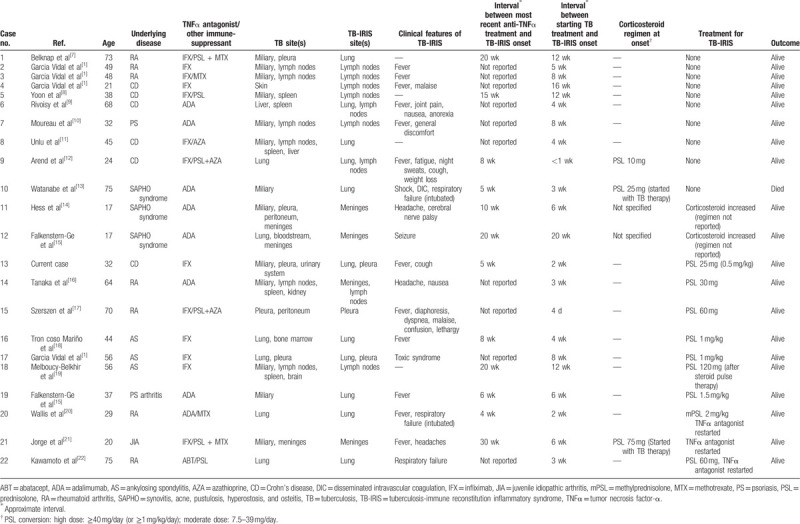
Summary of case reports of anti-tumor necrosis factor α-induced tuberculosis associated-immune reconstitution inflammatory syndrome.

Generally, treatment of tuberculous meningitis requires corticosteroids in addition to antituberculous therapy. Corticosteroids are also frequently used to treat acute respiratory failure in individuals with TB.^[23,24]^ In our review, 6 of the 12 patients (50%) receiving corticosteroids had meningitis or respiratory failure at the time of IRIS onset and were therefore considered to have severe TB-IRIS. A systemic steroid is recommended as supplemental treatment for HIV-associated TB-IRIS,^[25,26]^ so using corticosteroids should be considered in severe cases of anti-TNFα-induced TB-IRIS.

Conversely, corticosteroids can have an adverse effect on infectious diseases by inhibiting immune responses against pathogens in a dose-dependent manner.^[27]^ A meta-analysis showed that using corticosteroids in combination with antituberculous drugs did not always result in a better clinical outcome compared with using antituberculous drugs alone.^[28]^ Therefore, some physicians may avoid the use of corticosteroids for the treatment of TB-IRIS, or provide as low a dose as possible. In our review, high-dose corticosteroids (≥40 mg/day or ≥1 mg/kg/day) were not always necessary. Two cases, including the case presented in this report, were treated with moderate-dose corticosteroids (7.5–39 mg/day), and corticosteroids were not used in 8 (36%) of the cases that we reviewed. Therefore, our review suggests that cases of anti-TNFα-induced TB-IRIS are not always severe, and corticosteroids may not be required. Given these findings, further discussions are needed to determine indications for corticosteroid use, and the optimal dose for treating anti-TNFα-induced TB-IRIS.

Three cases of severe anti-TNFα-induced TB-IRIS included in this review restarted treatment with TNFα antagonists, and their anti-TNFα-induced TB-IRIS subsequently resolved.^[20–22]^ However, clinical data regarding restarting TNFα antagonists in patients with anti-TNFα-induced TB-IRIS is limited. TNFα antagonists have also been used to treat patients with steroid-refractory central nervous system TB, and can reduce the excessive inflammatory response to *M. tuberculosis* antigens and prevent IRIS.^[29,30]^ Although resuming treatment with TNFα antagonists in patients with anti-TNFα induced TB-IRIS may be therapeutic, resuming treatment with TNFα antagonists must be carefully considered because of TNFα's critical role in controlling TB. If TNFα antagonists can be used to shorten the use of high-dose steroid therapy, they may provide a good alternative treatment for anti-TNFα induced TB-IRIS. Rivoisy et al^[3]^ recommended that treatment with TNFα antagonists be resumed in severe cases of anti-TNFα-induced TB-IRIS, and Tanaka et al^[16]^ suggested that continuing TNFα antagonists may be effective for preventing anti-TNFα-induced TB-IRIS.

Among the cases that we reviewed, interval between the last administration of infliximab and the onset of IRIS in infliximab-associated cases (median: 6 weeks, range: 4–20 weeks) was longer than that of cases treated with other TNFα antagonists (median: 15 weeks, range: 5–30 weeks); however, this difference was not statistically significant (*P* = .22). This difference in the time of onset has been noted previously, and may be due to differences in the immune-recovery kinetics after anti-TNFα therapy has been discontinued.^[9]^

In conclusion, we successfully managed a patient with anti-TNFα-induced TB-IRIS using moderate-dose corticosteroids. The review of other case reports suggests that high-dose corticosteroids and/or TNFα antagonists may be needed for treating severe cases of anti-TNFα induced TB-IRIS, but that some mild cases may not require treatment. Low-to-moderate doses of corticosteroids appear to be effective in treating most mild cases. However, as corticosteroids were not used for half of the cases that we reviewed, the use of corticosteroids should be carefully considered in patients with anti-TNFα-induced TB-IRIS that do not experience spontaneous remission. Due to the limited evidence currently available, physicians should consider the necessity, dosage, and duration of corticosteroids for each case of anti-TNFα induced TB-IRIS on an individual patient-by-patient basis.

## Acknowledgment

We would like to thank Editage (www.editage.com) for English language editing.

## Author contributions

All authors contributed to the study conception and design. Material preparation, data collection, and analysis were performed by Daijiro Nabeya, Takeshi Kinjo, and Kazutaka Yamaniha. The first draft of the manuscript was written by Daijiro Nabeya. All authors provided comments on subsequent drafts of the manuscript, and read and approved the final draft.

## References

[1] Garcia Vidal C Rodríguez Fernández S Martínez Lacasa J Paradoxical response to antituberculous therapy in infliximab-treated patients with disseminated tuberculosis. Clin Infect Dis 2005;40:756–9. 15714425 10.1086/427941

[2] Dhasmana DJ Dheda K Ravn P Immune reconstitution inflammatory syndrome in HIV-infected patients receiving antiretroviral therapy: pathogenesis, clinical manifestations and management. Drugs 2008;68:191–208. 18197725 10.2165/00003495-200868020-00004

[3] Rivoisy C Tubach F Roy C Paradoxical anti-TNF-associated TB worsening: frequency and factors associated with IRIS. Joint Bone Spine 2016;83:173–8. 26677996 10.1016/j.jbspin.2015.04.022

[4] Namale PE Abdullahi LH Fine S Paradoxical TB-IRIS in HIV-infected adults: a systematic review and meta-analysis. Future Microbiol 2015;10:1077–99. 26059627 10.2217/fmb.15.9

[5] Bourgarit A Carcelain G Martinez V Explosion of tuberculin-specific Th1-responses induces immune restoration syndrome in tuberculosis and HIV co-infected patients. AIDS 2006;20:F1–7. 16511406 10.1097/01.aids.0000202648.18526.bf

[6] Sun HY Singh N Immune reconstitution inflammatory syndrome in non-HIV immunocompromised patients. Curr Opin Infect Dis 2009;22:394–402. 19483618 10.1097/QCO.0b013e32832d7aff

[7] Belknap R Reves R Burman W Immune reconstitution to Mycobacterium tuberculosis after discontinuing infliximab. Int J Tuberc Lung Dis 2005;9:1057–8. 16158902

[8] Yoon YK Kim JY Sohn JW Paradoxical response during antituberculous therapy in a patient discontinuing infliximab: a case report. J Med Case Rep 2009;3:6673. 19830122 10.1186/1752-1947-3-6673 PMC2726479

[9] Rivoisy C Amrouche L Carcelain G Paradoxical exacerbation of tuberculosis after TNFα antagonist discontinuation: beware of immune reconstitution inflammatory syndrome. Joint Bone Spine 2011;78:312–5. 21334948 10.1016/j.jbspin.2011.01.003

[10] Moureau C Pothen L Wilmes D Paradoxical response to tuberculosis treatment in a patient receiving tumor necrosis factor-alpha antagonist. Am J Med 2012;125:e9–10. 10.1016/j.amjmed.2012.02.006 22502953

[11] Unlu M Cimen P Ayranci A Disseminated tuberculosis infection and paradoxical reaction during antimycobacterial treatment related to TNF-alpha blocker agent Infliximab. Respir Med Case Rep 2014;13:43–7. 26029559 10.1016/j.rmcr.2014.09.005 PMC4246353

[12] Arend SM Leyten EM Franken WP A patient with de novo tuberculosis during anti-tumor necrosis factor-alpha therapy illustrating diagnostic pitfalls and paradoxical response to treatment. Clin Infect Dis 2007;45:1470–5. 17990230 10.1086/522993

[13] Watanabe S Kaneko Y Kawamoto H Paradoxical response with increased tumor necrosis factor-( levels to anti-tuberculosis treatment in a patient with disseminated tuberculosis. Respir Med Case Rep 2017;20:201–4. 28331797 10.1016/j.rmcr.2017.02.011 PMC5345969

[14] Hess S Hospach T Nossal R Life-threatening disseminated tuberculosis as a complication of TNF-α blockade in an adolescent. Eur J Pediatr 2011;170:1337–42. 21625932 10.1007/s00431-011-1501-y

[15] Falkenstern-Ge RF Husemann K Kohlhäufl M Prolonged paradoxical reaction to anti-tuberculous treatment after discontinuation of TNF-alpha- blocker therapy with adalimumab. Open Med (Wars) 2015;10:39–43. 28352675 10.1515/med-2015-0009 PMC5152954

[16] Tanaka T Sekine A Tsunoda Y Central nervous system manifestations of tuberculosis-associated immune reconstitution inflammatory syndrome during adalimumab therapy: a case report and review of the literature. Intern Med 2015;54:847–51. 25832955 10.2169/internalmedicine.54.2828

[17] Szerszen A Gupta S Seminara D Peritoneal tuberculosis complicated by immune reconstitution inflammatory syndrome in a patient treated with infliximab? A case for adjuvant immunosuppressive therapy. J Clin Rheumatol 2009;15:417–8. 19956004 10.1097/RHU.0b013e3181bc9407

[18] Troncoso Mariño A Campelo Sánchez E Martínez López de Castro N Haemophagocytic syndrome and paradoxical reaction to tuberculostatics after treatment with infliximab. Pharm World Sci 2010;32:117–9. 20127170 10.1007/s11096-010-9369-x

[19] Melboucy-Belkhir S Flexor G Stirnemann J Prolonged paradoxical response to anti-tuberculous treatment after infliximab. Int J Infect Dis 2010;14: suppl 3: e333–4. 20579914 10.1016/j.ijid.2010.03.002

[20] Wallis RS van Vuuren C Potgieter S Adalimumab treatment of life-threatening tuberculosis. Clin Infect Dis 2009;48:1429–32. 19364287 10.1086/598504

[21] Jorge JH Graciela C Pablo AP A life-threatening central nervous system-tuberculosis inflammatory reaction nonresponsive to corticosteroids and successfully controlled by infliximab in a young patient with a variant of juvenile idiopathic arthritis. J Clin Rheumatol 2012;18:189–91. 22647865 10.1097/RHU.0b013e318258b725

[22] Kawamoto H Takasaki J Ishii S Re-administration of abatacept for the control of articular symptoms of rheumatoid arthritis during anti-tuberculous therapy. Respir Med Case Rep 2017;21:147–50. 28507894 10.1016/j.rmcr.2017.04.013 PMC5423351

[23] Kim YJ Pack KM Jeong E Pulmonary tuberculosis with acute respiratory failure. Eur Respir J 2008;32:1625–30. 18614559 10.1183/09031936.00070907

[24] Yang JY Han M Koh Y Effects of corticosteroids on critically ill pulmonary tuberculosis patients with acute respiratory failure: a propensity analysis of mortality. Clin Infect Dis 2016;63:1449–55. 27609755 10.1093/cid/ciw616

[25] American Thoracic Society, CDC. Infectious Diseases Society of America. Treatment of tuberculosis. MMWR Recomm Rep 2003;52 (RR-11):1–77. 12836625

[26] Narita M Ashkin D Hollender ES Paradoxical worsening of tuberculosis following antiretroviral therapy in patients with AIDS. Am J Respir Crit Care Med 1998;158:157–61. 9655723 10.1164/ajrccm.158.1.9712001

[27] Stuck AE Minder CE Frey FJ Risk of infectious complications in patients taking glucocorticosteroids. Rev Infect Dis 1989;11:954–63. 2690289 10.1093/clinids/11.6.954

[28] Critchley JA Young F Orton L Corticosteroids for prevention of mortality in people with tuberculosis: a systematic review and meta-analysis. Lancet Infect Dis 2013;13:223–37. 23369413 10.1016/S1473-3099(12)70321-3

[29] Blackmore TK Manning L Taylor WJ Therapeutic use of infliximab in tuberculosis to control severe paradoxical reaction of the brain and lymph nodes. Clin Infect Dis 2008;47:e83–5. 18840076 10.1086/592695

[30] Lee HS Lee Y Lee SO Adalimumab treatment may replace or enhance the activity of steroids in steroid-refractory tuberculous meningitis. J Infect Chemother 2012;18:555–7. 22045163 10.1007/s10156-011-0334-y

